# Supplementary material for: The land flatworm Amaga expatria (Geoplanidae) in Guadeloupe and Martinique: new reports and molecular characterization including complete mitogenome
Source: PeerJ. 2020 Nov 9;8:e10098. doi: 10.7717/peerj.10098 (PMC7659627; doi:10.7717/peerj.10098)
Supplement: Supplemental Information 4 [file peerj-08-10098-s004.pdf]

Le Plathelminthe terrestre *Amaga expatria* (Geoplanidae) en Guadeloupe et Martinique: nouveaux signalements et caractérisation moléculaire, dont le mitogénome complet

Traduction de:

The land flatworm *Amaga expatria* (Geoplanidae) in Guadeloupe and Martinique: new reports and molecular characterization including complete mitogenome

PeerJ, 2020, 8:e10098

https://doi.org/10.7717/peerj.10098

Jean-Lou Justine [1]*, Delphine Gey [2], Jessica Thévenot [3], Romain Gastineau [4] and Hugh D. Jones [5]

[1] ISYEB - Institut de Systématique, Évolution, Biodiversité, Muséum National d'Histoire Naturelle, Paris, France

[2] Molécules de Communication et Adaptation des Micro-Organismes, Muséum National d'Histoire Naturelle, Paris, France

[3] Patrinat, Muséum National d'Histoire Naturelle, Paris, France

[4] Institute of Marine and Environmental Sciences, University of Szczecin, PL-70-383 Szczecin, Poland

[5] Scientific Associate, Life Sciences Department, Natural History Museum, London, SW7 5BD, UK.

* Auteur de correspondance: justine@mnhn.fr

Résumé

Contexte. Le Plathelminthe terrestre *Amaga expatria* Jones & Sterrer, 2005 (Geoplanidae) a été décrit à partir de deux spécimens collectés aux Bermudes en 1963 et 1988 et n'a pas été signalé depuis 2005.

Méthodes. Grâce à un projet de science participative, nous avons reçu des observations sur le terrain, des photographies et des spécimens de la part de non-professionnels et de scientifiques locaux, en Martinique et en Guadeloupe. Nous avons barcodé des spécimens (COI) des deux îles et étudié l'histologie des organes reproducteurs d'un spécimen. Par séquençage de nouvelle génération, nous avons obtenu le mitogénome complet d'*A. expatria* et des informations sur ses proies à partir de l'ADN contaminant.

Résultats. Nous rapportons des signalements de 2006 à 2019 dans deux îles françaises de l'arc caribéen, la Guadeloupe (six signalements) et la Martinique (14 signalements), à partir de photographies issues de la science participative et de spécimens examinés. Un spécimen de Martinique a été étudié pour l'histologie des organes copulateurs et a été barcodé pour le gène COI ; son anatomie était similaire à l'holotype, confirmant ainsi l'identification de l'espèce. Le gène COI était identique pour plusieurs spécimens de Martinique et de Guadeloupe et différait de plus de 10% des espèces les plus proches ; la caractérisation moléculaire de l'espèce est ainsi possible par des techniques classiques de codes-barres moléculaires. Le mitogénome a une longueur de 14962 pb et contient 12 gènes codant pour les protéines, deux gènes d'ARNr et 22 gènes d'ARNt ; pour deux gènes codants, il n'a pas été possible de déterminer le codon de départ. Le mitogénome a été comparé aux quelques mitogénomes disponibles des Geoplanidae et le plus similaire était *Obama nungara*, une espèce d'Amérique du Sud. Une analyse de l'ADN contaminant dans le système digestif suggère qu'*A. expatria* se nourrit de mollusques terrestres, et les observations de la science citoyenne sur le terrain suggèrent que ses proies comprennent des mollusques et des vers de terre ; l'espèce pourrait donc constituer une menace pour la biodiversité des animaux du sol dans les Caraïbes.

Introduction

Le Plathelminthe terrestre *Amaga expatria* Jones & Sterrer, 2005 (Plathelminthes, Geoplanidae) a été décrit à partir de deux spécimens collectés aux Bermudes en 1963 et 1988 (Jones & Sterrer 2005). Il n'a pas été signalé depuis. Jones & Sterrer (2005) ont conclu que l'espèce était exotique aux Bermudes et probablement introduite d'Amérique du Sud puisque les autres membres du genre ont été collectés dans cette région, notamment la Colombie, le Pérou, le Chili, le Brésil, le Paraguay et l'Argentine (Ogren & Kawakatsu 1990) ; le genre *Amaga* Ogren & Kawakatsu, 1990 comprend actuellement 10 espèces (Grau et al. 2012).

En 2003, l'un de nous (JLJ) a entrepris un programme de science participative en France sur les vers plats exotiques. Des documents ont été reçus de manière inattendue d'autres contrées, y compris des territoires français d'outre-mer (Justine et al.2014, 2015, 2018a, 2018b, 2019 ; Justine & Winsor 2020). Parmi ceux-ci figuraient plusieurs signalements de grands vers plats terrestres des deux îles des Caraïbes, la Martinique et la Guadeloupe, avec des dimensions et une pigmentation similaires à *A. expatria* et provisoirement identifiés comme tels. Pour confirmer cette identification, un spécimen de Martinique a été partiellement sectionné pour comparaison anatomique avec le matériel type. Le même échantillon a également été soumis à un séquençage moléculaire (barcode COI). Un autre spécimen de Martinique a été inclus dans une étude comparative du mitogénome de plusieurs vers plats terrestres (Gastineau & Justine 2020 ; Gastineau et al. 2019, 2020).

Nous présentons ici de nouveaux signalements d'*A. expatria* dans deux îles des Caraïbes, et fournissons des informations morphologiques supplémentaires, la première caractérisation par codes-barres de l'espèce et son mitogénome complet.

Matériels et méthodes

Science participative et collecte d'informations

Les signalements ont été collectés de 2013 à 2019, sur une période de 7 ans (des signalements uniques de 2006 et 2012 sont également inclus). Nous avons utilisé les mêmes méthodes que pour nos recherches précédentes sur les vers plats terrestres (Justine et al. 2014, 2015, 2018a, 2018b, 2019). Un blog (Justine 2019) et un compte Twitter (@ Plathelminthe4) étaient les

principaux outils de collecte et de transmission d'informations. La collaboration des associations locales d'histoire naturelle et des FREDON (Fédérations Régionales de Défense contre les Organismes Nuisibles) dans les départements de la Martinique et de la Guadeloupe a également été précieuse. Des rapports d'observations ont été reçus du grand public et de professionnels, généralement par courrier électronique. Nous avons sollicité et obtenu des spécimens. Les spécimens ont été obtenus vivants, fixés dans de l'eau presque bouillante et conservés dans de l'éthanol à 95%, ou parfois fixés directement dans de l'éthanol froid. Ils ont été envoyés au Muséum National d'Histoire Naturelle (MNHN) à Paris, enregistrés et traités pour des études moléculaires.

Histologie

Un spécimen de Martinique, MNHN JL146, a été utilisé pour l'histologie. Il a été tué vivant dans de l'eau bouillante, puis conservé dans de l'éthanol à 80%. Une partie d'environ 1,7 cm de long contenant l'appareil copulateur a été retirée pour la coupe. Des coupes longitudinales horizontales ont été coupées à une épaisseur de 12 µm, montées sur 41 lames, colorées à l'hématoxyline et à l'éosine et montées en baume du Canada (les lames 1-5 et 38-41 restent non colorées dans la cire). Les lames sont déposées au MNHN, Paris, numéro d'enregistrement MNHN JL146.

Barcoding moléculaire

Pour l'analyse moléculaire, un petit morceau du corps (1 à 3 mm^3) a été prélevé sur le bord latéral d'individus fixés à l'éthanol. L'extraction de l'ADN et la PCR ont été réalisées comme dans des travaux similaires précédents (Justine et al. 2019). En bref, un fragment de 424 pb a été amplifié avec les amorces JB3 (= COI-ASmit1) et JB4.5 (= COI-ASmit2) (Bowles et al. 1995 ; Littlewood et al. 1997), et un fragment de 825 pb a été amplifié avec les amorces BarS (Álvarez-Presas et al. 2011) et COIR (Lázaro et al. 2009 ; Mateos et al. 2013). Les produits de PCR ont été purifiés et séquencés dans les deux sens sur un séquenceur d'ADN 3730xl à 96 capillaires (Applied Biosystems). Les résultats des deux analyses ont été concaténés pour obtenir une séquence COI de 903 pb de longueur. Les séquences ont été vérifiées en utilisant le logiciel CodonCode Aligner (CodonCode Corporation, Dedham, MA, USA), comparées au contenu de la base de données GenBank en utilisant BLAST, et déposées dans GenBank sous les numéro d'accès MT602619-MT602626.

Séquençage de nouvelle génération, phylogénie et identification de l'ADN contaminant

Un morceau des tissus du spécimen MNHN JL305 a été envoyé au Beijing Genomics Institute (BGI) à Shenzhen, qui a assuré l'extraction et le séquençage de l'ADN. Le séquençage a été réalisé sur une plateforme DNBSEQ. Un total d'environ 60 millions de lectures appariées propres ont été obtenues. Les lectures ont été assemblées à l'aide de SPAdes 3.14.0 (Bankevich et al. 2012) avec un k-mer de 85. Les contigs correspondant au mitogénome et aux gènes de l'ARN ribosomal nucléaire ont été récupérés par des analyses de ligne de commande blastn personnalisées, en utilisant des séquences déjà disponibles téléchargées depuis GenBank comme base de données personnalisée ; les gènes d'ARN ribosomal (non utilisés dans cet article) ont été déposés dans GenBank sous les numéros d'enregistrements MT860713 (18S) et MT860719 (28S partiel). En plus des séquences liées à *A. expatria*, d'autres correspondances positives appartenant à l'ADN contaminant ont été obtenues, comme expliqué dans les résultats. Les ARNt ont été identifiés à l'aide d'un scan ARNt (Lowe & Chan 2016). Le mitogénome a été vérifié à l'aide du package Consed (Gordon et al. 1998), et l'identification des gènes a été réalisée à l'aide de MITOS (Bernt et al. 2013). La carte génomique a été établie à l'aide de OGDRAW (Lohse et al. 2013). Les séquences d'acides aminés des gènes codant pour la protéine ont été concaténées suivant un protocole déjà décrit (Gastineau & Justine 2020 ; Gastineau et al. 2019, 2020), et alignées avec les séquences correspondantes d'autres espèces en utilisant MAFFT (Katoh & Standley 2013) ; nous avons utilisé la version en ligne de commande de MAFFT, avec l'option «G-INS-I». Une phylogénie du maximum de vraisemblance a été déduite de cet alignement en utilisant RaxML version 8.0 (Stamatakis 2014) en utilisant le modèle de substitution MtArt (Abascal et al. 2007). Le meilleur arbre sur 100 a été calculé pour 100 répliques bootstrap.

Arbres COI et distances

MEGA7 (Kumar et al.2016) a été utilisé pour évaluer les distances et construire des arbres. Pour le groupe externe, nous avons choisi une séquence dans GenBank de l'espèce sud-américaine *O. nungara* Carbayo, Álvarez-Presas, Jones & Riutort, 2016 (Carbayo et al. 2016) (MN529572) qui avait une couverture de requête à 100% avec nos séquences.

Résultats

Informations obtenues grâce à la science participative et d'autres scientifiques

Nous avons obtenu 14 signalements vérifiés de la Martinique (carte de la Figure 1), de 2006 à 2018, et six signalements vérifiés de Guadeloupe, de 2012 à 2018 (carte de la Figure 2). Les signalements ont généralement été obtenus sous forme de photographies, mais nous avons également reçu cinq spécimens de Martinique et trois spécimens de Guadeloupe (Tableau 1). Par ailleurs, François Meurgey (courriel, 29.01.2016) a ajouté des informations sur la Guadeloupe : « *Amaga expatria* est assez commun dans la haute série de la forêt mésophile et entre 400 et 700 m d'altitude dans la forêt humide. Je l'ai rencontré dans les communes de Baillif (rivière St Louis), Matouba, Trois-Rivières et Gourbeyre. Il semble plus fréquent dans le sud de la Basse-Terre. Je l'ai observé attaquant des escargots des genres *Helicina*, *Pleurodonte* et des vers de terre ». Laurent Charles (courriel, 15.05.2020) a envoyé une photographie montrant la prédation sur un escargot identifié comme *Helicina platychila* (Megerle von Mühlfeld, 1824).

Morphologie et histologie

Les spécimens vivants mentionnés dans cette étude, mesurés sur les photographies (Figures 3 à 5), mesuraient 128 à 132 mm de longueur et 5,5 à 9 mm de largeur à l'état étendu et 35 × 12 mm à l'état contracté.

Description de l'échantillon MNHN JL146.

Mesures sur le vivant (Figure 3) : longueur 128 mm, largeur 5,5 mm. Mesures de l’animal préservé : longueur 108 mm, largeur 9 mm, hauteur 2 mm ; bouche 53% ; pore génital 75%.

L'appareil copulateur (Figure 6) mesure environ 8 mm de long de la partie antérieure du système mâle à la partie postérieure du système femelle. Le système mâle mesure environ 5 mm de long et le système femelle environ 3 mm de long.

Deux canaux spermatiques, distants d'environ 1,2 mm, chacun avec du sperme stocké abondant (cyanophile) s'approchent de l'appareil copulateur, tournent brièvement en avant, avant de s'ouvrir séparément dans l'extrémité ventrale d'un seul canal (Figure 6d). Ce canal a une paroi musculaire épaisse et s'étend verticalement de la face ventrale à la dorsale sur environ 1080 µm (présents dans 90 sections × 12 µm). À son extrémité dorsale, ce canal se prolonge vers l'arrière

sous la forme d'un canal sinueux étroit avant de s'élargir dans le canal éjaculateur. Le canal éjaculateur se termine par un pénis court d'environ 800 µm de long et 600 µm de large (Figure 6a, b). Il y a des plis auriculaires à l'extérieur du pénis dans l'antre commun.

Les deux canaux ovovitellins (Figure 6a, b) sont distants d'environ 2 mm en avant du pénis, ils courent en arrière et à peu près au niveau du pénis, tournent dorsalement pour se rejoindre et s'ouvrir dans le canal femelle combiné. Des glandes coquillières abondantes (éosinophiles) sont présentes et s'ouvrent dans les deux canaux ovovitellins avant de se joindre (Figure 6c) pour former le canal femelle combiné. Le canal femelle combiné s'élargit et présente un ou deux plis longitudinaux, avant de s'ouvrir dans l'antre commun.

Le gonopore s'ouvre à partir de l'antre commun via un conduit court et étroit.

Caractérisation moléculaire - COI

Pour 4 échantillons, les séquences COI amplifiées obtenues étaient identiques sur toute leur longueur (903 pb). Ces spécimens étaient JL289, JL305 (obtenus à la fois du séquençage de Sanger et du mitogénome) et JL310 de Martinique, ainsi que JL319 de Guadeloupe. Cela démontre que la même espèce a été trouvée dans les deux îles. Le spécimen JL146 de Martinique, qui a été traité pour l'histologie, et trois autres spécimens, JL216 et JL217 de Guadeloupe et JL262 de Martinique, ont fourni des séquences plus courtes mais elles étaient également identiques entre elles et avec les 4 séquences ci-dessus pour leur portion en commun. Cela démontre que le spécimen étudié pour l'histologie est de la même espèce, confirmant ainsi que l'espèce dans les deux îles est *A. expatria*. La différence avec *O. nungara*, calculée sur les 903 pb en commun, était de 12%.

Mitogénome

Le mitogénome (Figure 7) a une longueur de 14962 pb (numéro d'accès GenBank : MT527191). Il contient 12 gènes codant pour les protéines, 2 gènes d'ARN ribosomal et 22 gènes d'ARN de transfert. Le mitogénome est complètement colinéaire avec celui d'*O. nungara* (KP208777) (Solà et al. 2015) et de taille similaire (14909 pb pour *O. nungara*). Une requête megablast utilisant la séquence entière du mitogénome montre une identité globale de 83,77% entre ces deux espèces. Le mitogénome est également colinéaire avec ceux de *Bipalium kewense*, mais pas avec ceux de *Platydemus manokwari* et *Parakontikia ventrolineata*. Pour 3 gènes, atp6, cox2

et ND3, il n'a pas été possible de déterminer le codon de départ. La première méthionine des protéines prédites survient en position 72/224 pour atp6, 112/260 pour cox2 et 44/112 pour ND3. Il convient de mentionner que l'impossibilité de mettre en évidence un codon de départ pour ces gènes a été observée avec *O. nungara*, mais pas par exemple avec *B. kewense*, *Pl. manokwari* ou *Pa. ventrolineata*. Contrairement à *O. nungara*, cependant, aucun chevauchement entre les gènes ND4L et ND4 n'a été mis en évidence.

Dans l'arbre représentant une phylogénie obtenue par maximum de vraisemblance des séquences d'acides aminés des gènes codant pour des protéines, *A. expatria* est le groupe-frère d'*O. nungara* (Figure 8).

Détection d'ADN contaminant

Après assemblage, les séquences liées à l'ADN contaminant ont été identifiées. Trois contigs de 3080 pb, 7274 pb et 15202 pb ont été récupérés. Les analyses Megablast ont été réalisées sur le portail NCBI. Les meilleurs résultats sont listés par la suite. Le fragment de 3080 pb présentait une identité de 99,66% avec les gènes ribosomaux 18S de séquences identifiées comme issues des mollusques *Subulina striatella* (Rang, 1831) (MN022690), *Lissachatina fulica* (MN022692) et *Achatina fulica* (Férussac, 1821) (KU365375). Le fragment de 7274 pb a montré une identité de 99,97% avec l'espaceur transcrit interne 2 d'une séquence identifiée comme *Subulina octona* (Bruguière, 1789) (MF444887). Le fragment le plus long semblait être un génome mitochondrial presque complet. Le gène cox1 en a été extrait, et il a montré une identité de 97,86% avec *S. octona* (JX988065).

Discussion

Nouveaux signalements

Amaga expatria a été décrit sur la base de deux spécimens des Bermudes, l'holotype, collecté en 1988, et un paratype, collecté en 1963 (Jones & Sterrer 2005). L'espèce n'a pas été répertoriée depuis, mais a été mentionnée dans un livre sur les mollusques de la Martinique (Delannoye et al. 2015) ; ces signalements ont été identifiés à l'origine par l'un de nous (JLJ) et sont également inclus dans la présente étude. Notre étude compte dix fois plus de signalements que la description originale, avec 6 signalements de Guadeloupe et 14 signalements de Martinique

(tableau 1). Cela illustre à nouveau le pouvoir de la science citoyenne pour inventorier les vers plats terrestres (Justine et al.2018b, 2019).

Les cartes (Figures 1-2) montrent que l'espèce est largement répandue en Martinique et en Guadeloupe. En Guadeloupe, la plupart des signalements provenaient de Basse Terre et un seul signalements (Sainte-Anne) provenait de Grande Terre, et en Martinique, la plupart des signalements provenaient du Nord de l'île, avec un seul au Sud, à Sainte-Luce. Cela suggère que l'espèce est plus abondante, mais non exclusivement, dans les parties des îles où les précipitations sont plus importantes (Basse Terre en Guadeloupe et le nord en Martinique).

Anatomie et morphologie

L'appareil copulateur du spécimen de Martinique (Figure 6) est essentiellement le même que celui du spécimen type d'*A. expatria* des Bermudes (NHMUK.2002.10.16.1). Les canaux mâles afférents ont une structure similaire, avec deux canaux de spermatozoïdes se déchargeant dans un canal ventro-dorsal qui à son tour débouche dans le canal éjaculateur et le pénis émoussé via un canal sinueux. La paroi du conduit est épaissie dans la même position à peu près à mi-chemin entre le conduit vertical et le pénis. Nous sommes convaincus que le spécimen de Martinique (MNHN JL146) doit être identifié comme *A. expatria*. Compte tenu de cela et de la similitude des caractéristiques externes des spécimens de Martinique, de Guadeloupe et des Bermudes, nous sommes convaincus que tous les spécimens sont *A. expatria*.

Régime alimentaire

L'analyse de l'ADN des proies est une méthode efficace pour déterminer le régime alimentaire des Plathelminthes terrestres (Cuevas-Caballé et al.2019). Toutes les analyses BLAST de l'ADN du contaminant dans un spécimen d'*A. expatria* l'ont identifié comme appartenant aux Gastropoda, et il est probable que la proie était un spécimen de *Subulina octona*, ou une espèce étroitement apparentée. *Subulina octona* est un escargot terrestre tropical, avec une distribution cosmopolite ; ce mollusque est en effet présent en Martinique où il est considéré comme récemment introduit (Anonyme 2020).

La description originale d'*A. expatria* n'incluait aucune observation directe sur le régime alimentaire, mais Jones et Sterrer (2005), sur la base de la présence d'un pharynx plissé, ont écrit : « il est probable que les vers de terre soient la seule ou principale proie d'*A. expatria* ».

Les observations de François Meurgey (prédation sur les escargots des genres *Helicina*, *Pleurodonte* et vers de terre) et Laurent Charles (prédation sur *Helicina platychila*) rapportées ici, et la découverte de la séquence d'un mollusque terrestre dans l'intestin, indiquent un régime généraliste, comprenant les mollusques et les vers de terre. Ce régime alimentaire pourrait être l'une des raisons du succès de l'espèce à envahir diverses îles des Caraïbes.

Barcoding moléculaire

Un spécimen de la Martinique a été caractérisé pour la morphologie, l'histologie et le code-barres et ce travail représente ainsi la première tentative de caractérisation moléculaire de l'espèce. Des spécimens de Martinique et de Guadeloupe ont fourni des séquences identiques, démontrant ainsi sans équivoque que la même espèce est présente sur les deux îles et que cette espèce, par la morphologie et l'anatomie, est *A. expatria*. L'absence de divergence génétique entre nos spécimens séquencés suggère que l'espèce a été récemment introduite dans les deux îles à partir d'une seule population. La séquence COI la plus proche d'*A. expatria* trouvée par BLAST était *O. nungara*, avec une différence significative de 12%. Cela suggère que les séquences COI peuvent être utilisées pour le barcoding d'*A. expatria*, mais cela devrait être validé à l'avenir par une étude comparative avec des séquences d'autres espèces d'*Amaga*, qui ne sont actuellement pas disponibles.

Génome mitochondrial et phylogénie multigénique

Dans la phylogénie multigénique par maximum de vraisemblance, le clade comprenant *A. expatria* et *O. nungara* a un fort support de nœud de 100, ce qui est en accord avec leur position dans la même sous-famille des Geoplaninae (Figure 8). Cela les distingue clairement du Bipaliinae *B. kewense*, du Caenoplaninae *Pa. ventrolineata* et du Rhynchodeminae *Pl. manokwari*.

Parmi les caractéristiques conservées entre les mitogénomes d'*A. expatria* et *O. nungara*, nous tenons à souligner l'absence conservée de codons de départ canoniques pour les trois gènes atp6, cox2 et ND3. Au lieu de cela, les premiers acides aminés mis en évidence par les protéines putatives sont toujours une leucine. Cette leucine est toujours codée par un codon TTG, sauf pour *A. expatria* où elle est remplacée par TTA. Bien que rien de tel n'ait été mis en évidence parmi les mitogénomes récemment séquencés de *B. kewense*, *Pl. manokwari* et *Pa.*

ventrolineata (Gastineau & Justine 2020 ; Gastineau et al. 2019, 2020), des caractéristiques similaires ont également été observées chez plusieurs Dugesiidae tels que *Dugesia japonica* AB618487, *Dugesia ryukyuensis* AB618488 (tous deux dans Sakai & Sakaizumi 2012), *Girardia* sp. KP090061 et *Schmidtea mediterranea* NC022448 et KM821047 (tous deux dans Ross et al. 2016). La possibilité que TTG puisse agir comme un codon de départ alternatif a déjà été suggérée par Ross et al. (2016). Sur la base de nos données, nous pouvons suggérer que TTA pourrait également être envisagé. Répondre correctement à cette question pourrait nécessiter un séquençage N-terminal de ces protéines.

Notons qu'*Amaga* et *Obama* appartiennent à la sous-famille des Geoplaninae, tandis que *Platydemus* et *Parakontikia* sont membres des Rhynchodeminae et *Bipalium* est membre des Bipaliinae. Cette éventuelle variation du code génétique pourrait donc être limitée à une seule sous-famille des Geoplanidae, les Geoplaninae.

Conclusion

Notre étude montre qu'une espèce de ver plat terrestre, de taille relativement grande, est commune dans deux îles des Caraïbes et, avec 20 nouvelles mentions, apporte dix fois le nombre précédent de signalements connus de l'espèce, qui provenaient d'un seul endroit, les Bermudes, une île située dans l'océan Atlantique Nord-Est. Jones & Sterrer (2005) ont émis l'hypothèse que l'espèce provenait de l'Amérique du Sud continentale et avait été récemment introduite aux Bermudes. Nos résultats génétiques montrent que les séquences COI de Martinique et de Guadeloupe sont identiques et suggèrent donc que l'introduction est récente dans ces îles. Il reste que la localité d'origine de l'espèce en Amérique du Sud est encore inconnue. L'espèce se nourrit de mollusques et de vers de terre et pourrait constituer une menace pour la biodiversité des animaux du sol, en particulier les mollusques qui comprennent des espèces endémiques et rares dans les îles des Caraïbes (Delannoye et al. 2015). Cependant, aucune prolifération n'a été enregistrée et la menace peut être mineure, mais il se peut aussi qu'*A. expatria* n'en soit qu'aux premiers stades de l'invasion et que cette espèce devienne une espèce envahissante à l'avenir ; de même, des observations récentes ont montré que l'espèce très invasive *Platydemus manokwari* envahit désormais la Guadeloupe (Justine & Winsor 2020). La présence d'*A. expatria* dans deux îles des Caraïbes suggère que l'espèce pourrait être présente dans d'autres îles, et peut-être en Amérique du Nord continentale.

Remerciements

Nous sommes reconnaissants à tous les non-professionnels et professionnels qui ont fourni des signalements et des spécimens ; ils sont énumérées dans le Tableau 1. Toutes les personnes énumérées dans le Tableau 1 ont aimablement accepté que leurs photographies soient publiées dans ce document. François Meurgey et Laurent Charles ont aimablement communiqué quelques observations de terrain ; Laurent Charles a fourni des informations sur le statut de *Subulina octona* ; ils ont tous deux accepté que leurs informations soient communiquées ici. Leigh Winsor (Université James Cook, Australie) a aimablement aidé à identifier les spécimens sur photographies aux premiers stades de ce travail.

Figures

Figure 1. *Amaga expatria*, carte des signalements en Martinique.

Les couleurs de fond indiquent les chutes de pluie annuelles. La plupart des signalements proviennent de la partie nord de l'île où la pluviométrie est élevée, mais le signalement de Sainte-Luce dans le sud provient d'une partie relativement plus sèche. Carte par Jessica Thévenot, fond fourni par Météo-France et utilisé avec autorisation.

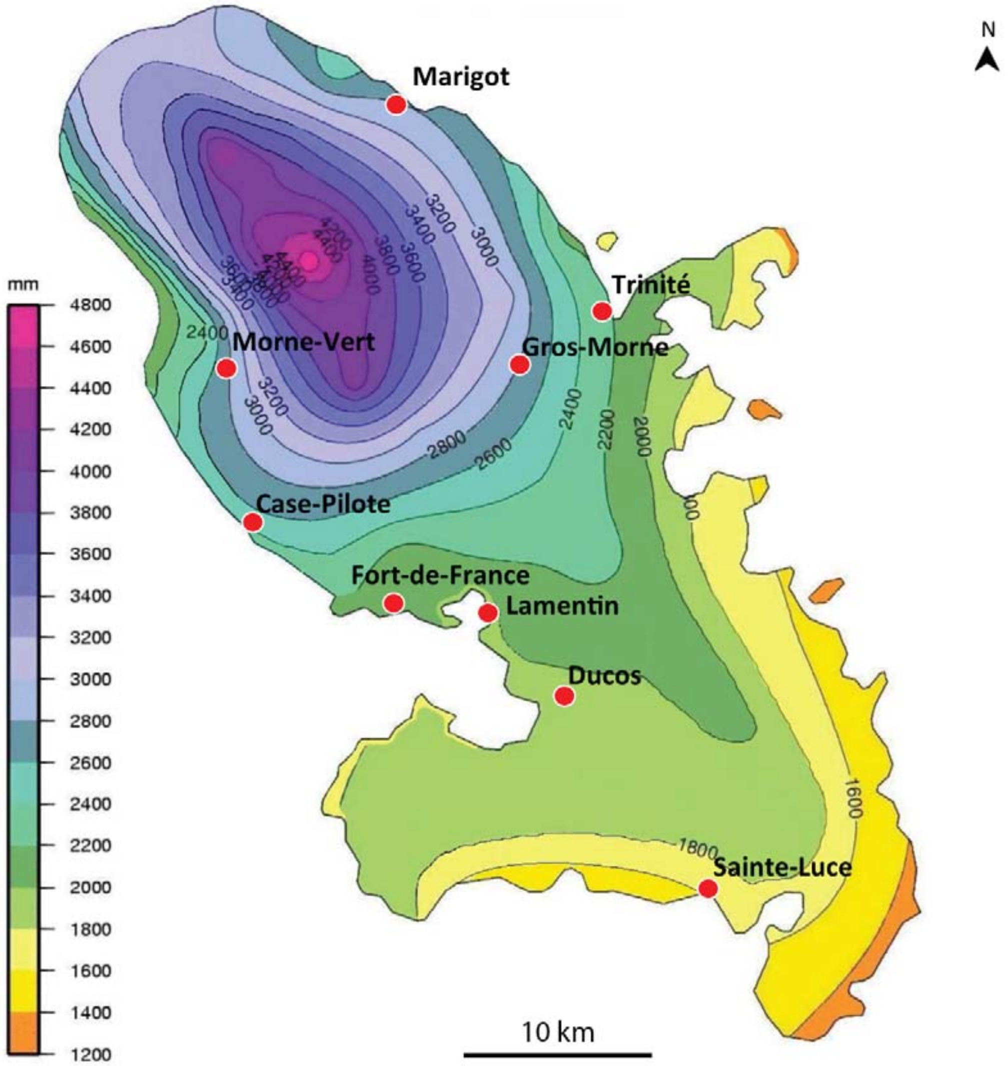

Figure 2. *Amaga expatria*, carte des signalements en Guadeloupe.

Les couleurs de fond indiquent les chutes de pluie annuelles. La plupart des signalements proviennent de Basse Terre, où la pluviométrie est élevée, mais le signalement de Ste-Anne à Grande Terre provient d'une région relativement plus sèche. Carte par Jessica Thévenot, fond fourni par Météo-France et utilisé avec autorisation.

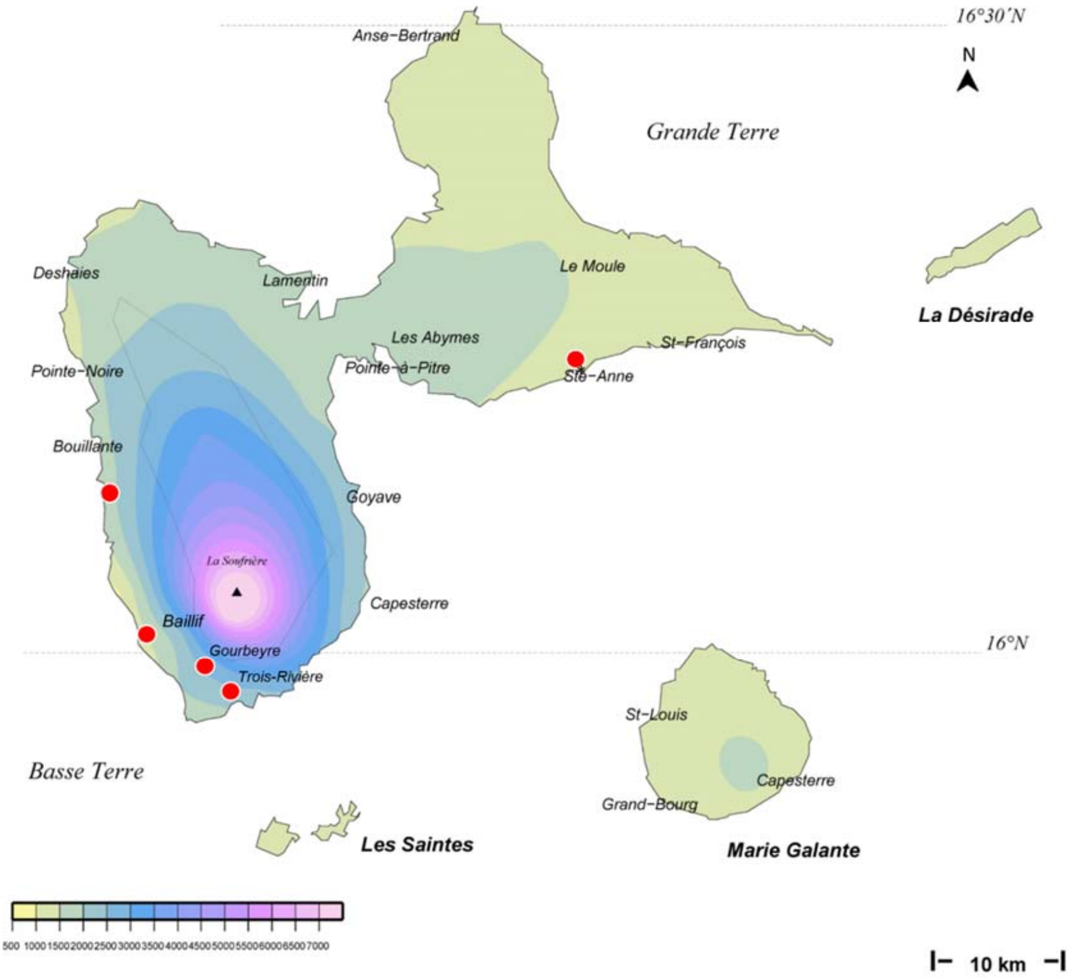

Figure 3. ***Amaga expatria*, spécimen MNHN JL146 de Martinique.**

A, spécimen vivant, photographie par Clément Dromer; échelle: le diamètre de la pièce est de 24 mm; B, spécimen conservé, photographie par Jean-Lou Justine. Ce spécimen a été utilisé pour l'anatomie.

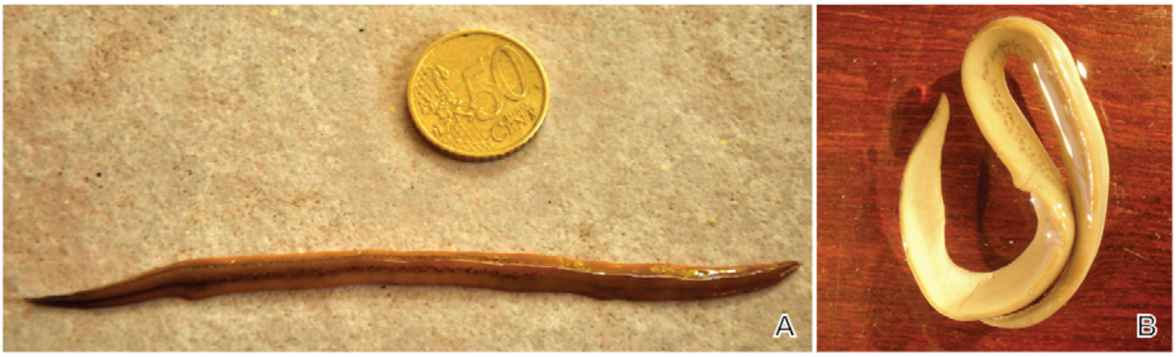

Figure 4. ***Amaga expatria* de Martinique, spécimens vivants.**

A, photographie par Cédric Rareg; B, C Photographie par Régis Delannoye; C, échelle en mm; D, photographie par Mathieu Coulis, spécimen MNHN JL305.

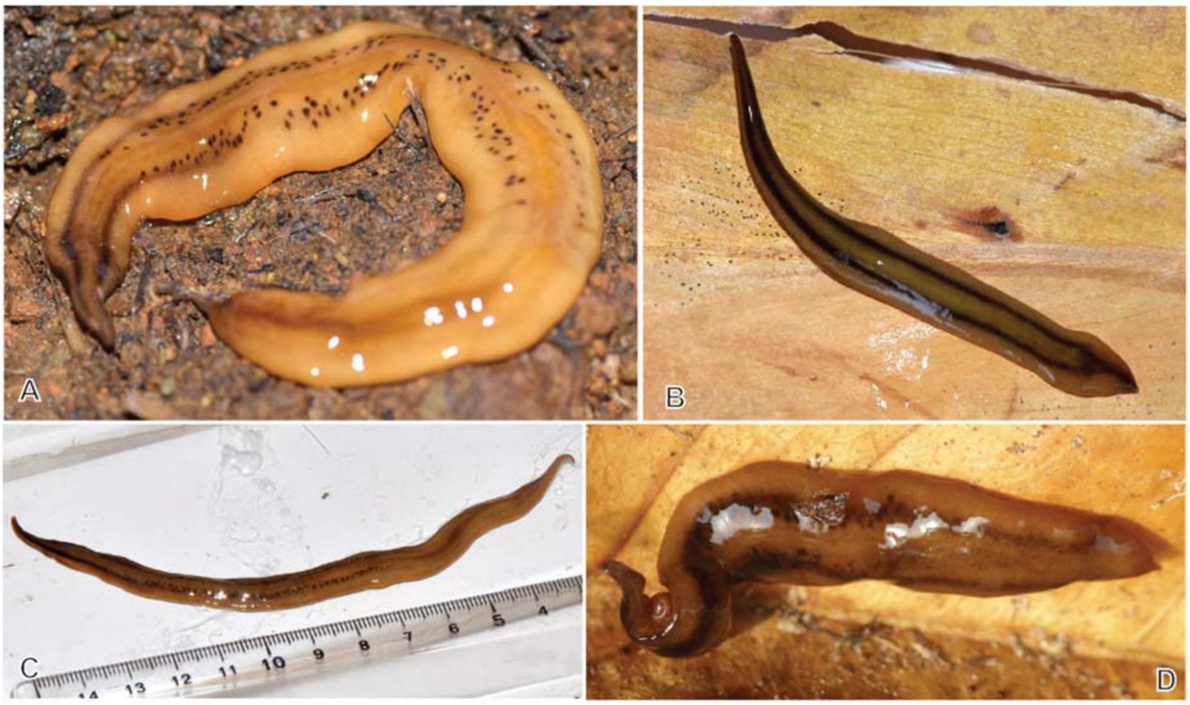

Figure 5. ***Amaga expatria*** **de Guadeloupe, spécimens vivants sur le terrain.**

A, photographie par Pierre et Claude Guezennec; B, C, photographies par Laurent Charles, B, spécimen MNHN JL216, C, MNHN JL217; l'escargot proie est *Helicina platychila*; D, photographie par Mathieu Coulis, spécimen MNHN JL310; E-G, photographies par Guy van Laere, E, F, spécimen MNHN JL319, E, face dorsale, F, montrant la face ventrale.

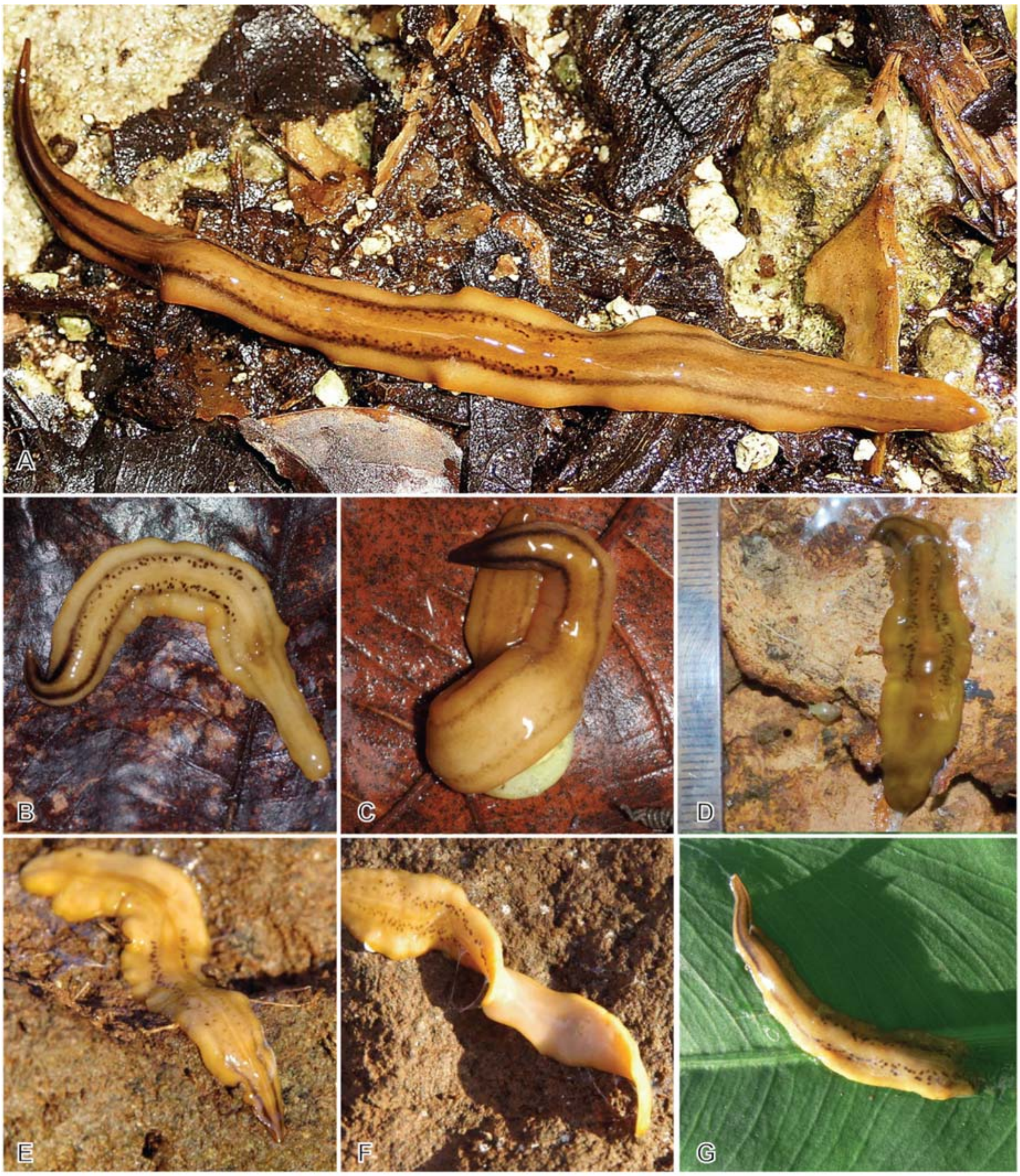

Figure 6. *Amaga expatria*, spécimen MNHN JL146 de Martinique, anatomie.

Anatomie de l'appareil copulateur, l'avant est à droite: A & B respectivement une coupe HLS et une reconstruction schématique à travers l'appareil copulatoire à la même échelle; C, partie postérieure des canaux femelles montrant la jonction des canaux ovovitellins; D, partie antérieure des canaux mâles montrant la jonction des canaux spermatiques, à la fois avec un abondant sperme stocké (cyanophile) et avec l'extrémité ventrale du canal spermatique vertical. Échelle 2 mm en A, 0,5 mm en C et D. Abréviations: ed, canal éjaculateur; jod, jonction des canaux ovovitellins; jsd, jonction des canaux spermatiques et du canal spermatique vertical; od, canal ovovitellin; p, pénis; sd, canal spermatique; shg, glande coquillère; vsd, canal spermatique vertical.

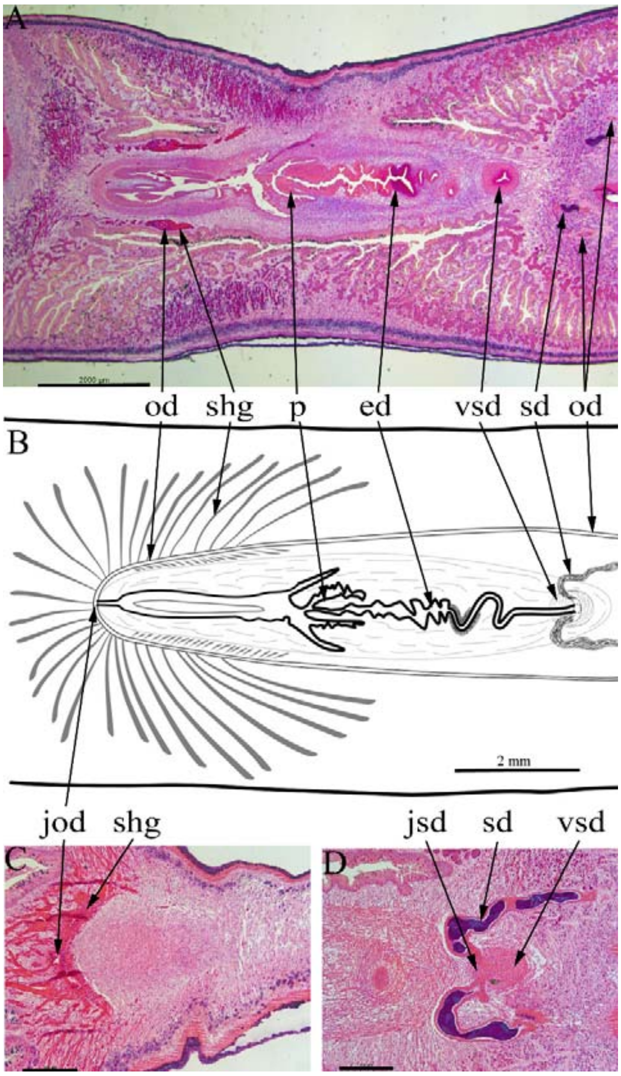

Figure 7. *Amaga expatria*, carte génomique du génome mitochondrial.

Le mitogénome a une longueur de 14 962 pb et contient 12 gènes codant pour les protéines, 2 gènes d'ARN ribosomique et 22 gènes d'ARN de transfert. Pour 3 gènes, atp6, cox2 et ND3, il n'a pas été possible de déterminer le codon de départ.

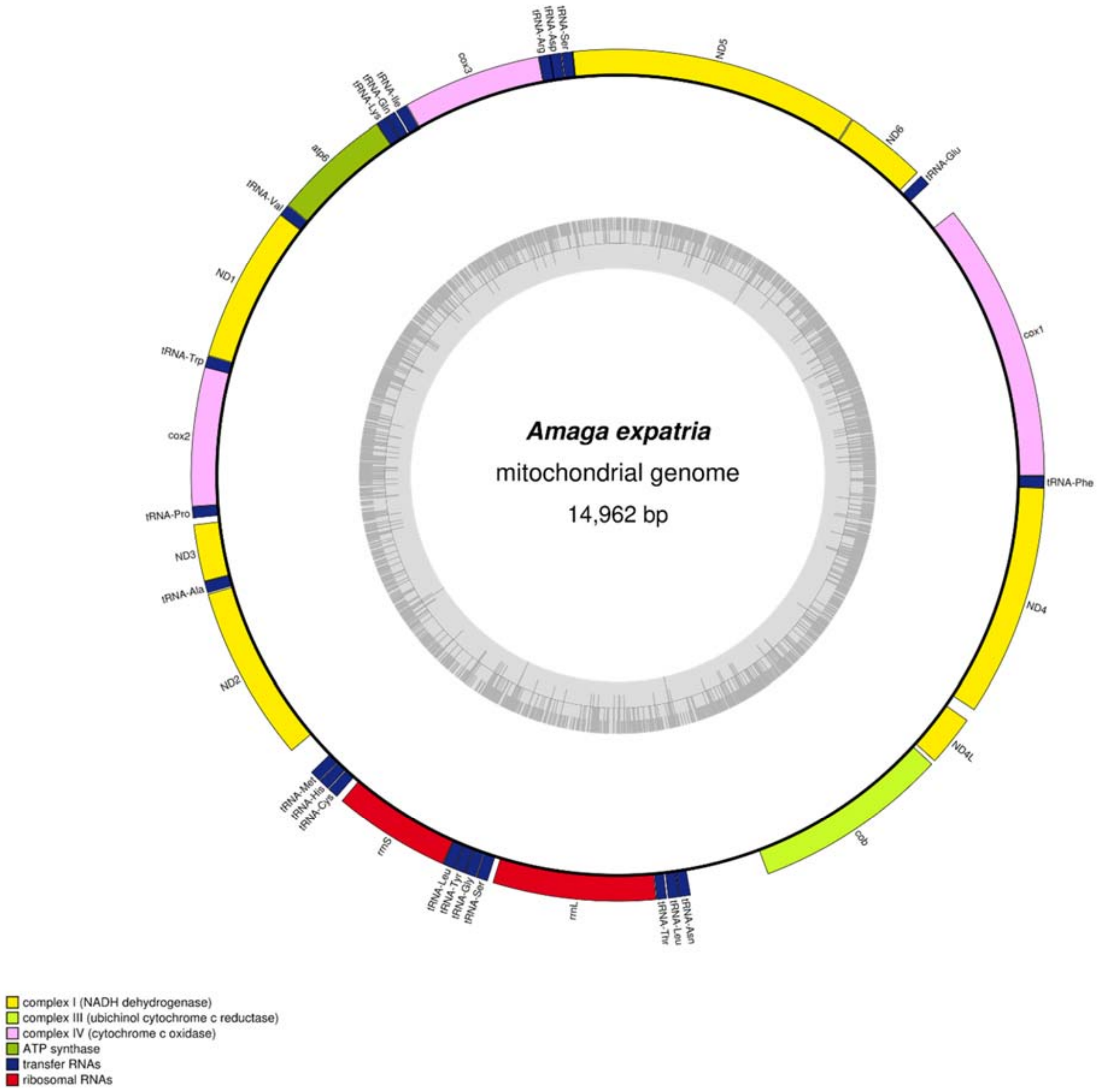

Figure 8. Arbre de maximum de vraisemblance des protéines des mitogénomes.

Les protéines des mitogénomes ont été obtenues à partir de séquences d'acides aminés concaténées de tous les gènes codant pour les protéines mitochondriales d'*Amaga expatria* et d'autres Plathelminthes. Arbre obtenu en utilisant le modèle d'évolution MtArt après 100 réplications bootstrap. L'arbre avec la meilleure probabilité est affiché (-62178,796969).

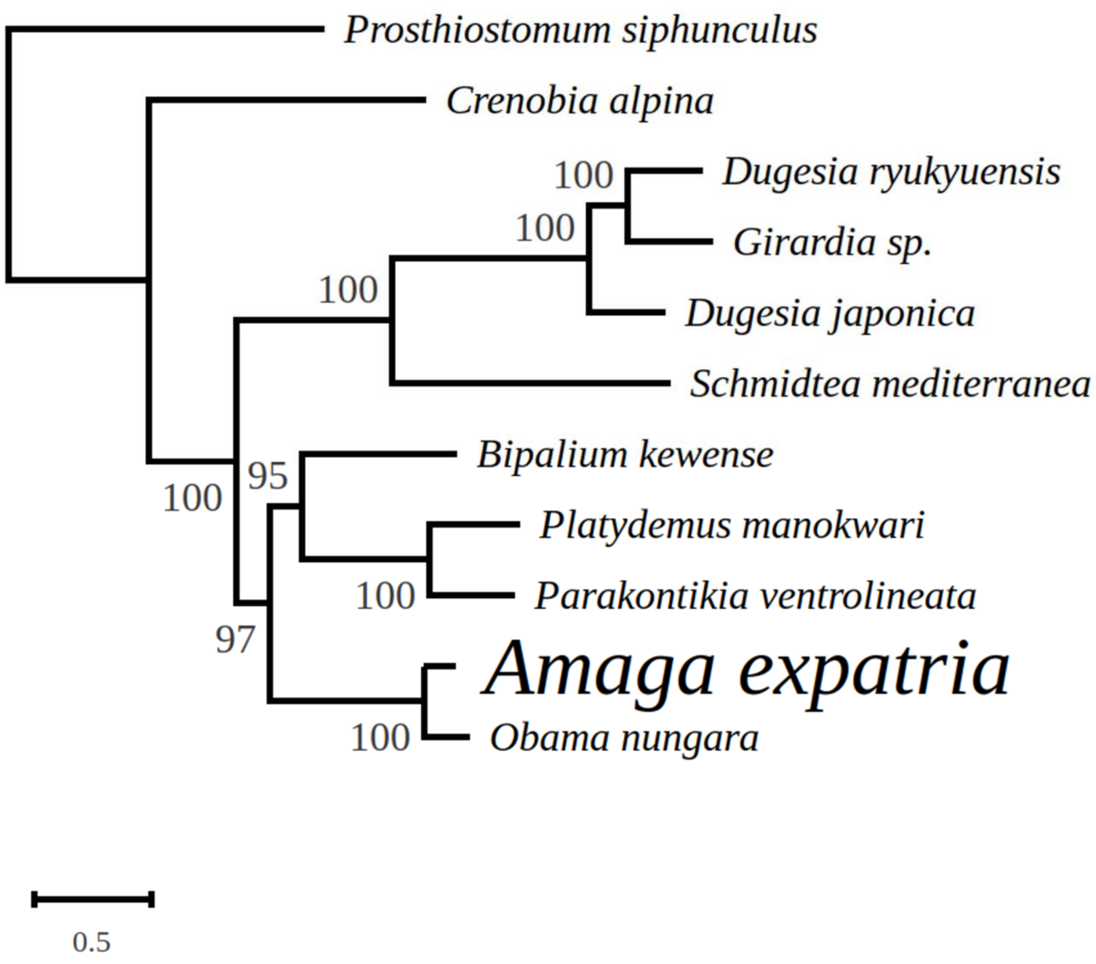

Tableau 1. Signalements d'*Amaga expatria* en Guadeloupe et Martinique.

Le tableau ne comprend que des observations basées sur des photographies et des spécimens. François Meurgey a fourni des résultats complémentaires en Guadeloupe: 10.10.2019, Goyave; 16.10.2019, Vieux-Fort; 22.06.2019, Petit Canal.

* Pour le MNHN JL305, nous avons obtenu à la fois une séquence COI (GenBank MT602624) et le mitogénome complet (GenBank MT527191).

Date	Signalement (numéro de spécimen et/ou photo)	Nombre de spécimens	Commune	Département	Séquence COI	Collecteur/ Observateur
28/02/2006	photo	0	Case-Pilote	Martinique	non	Régis Delannoye
17/08/2013	photo	0	Case-Pilote	Martinique	non	Régis Delannoye
25/11/2013	film	0	Fort de France	Martinique	non	Anonymous
20/12/2013	photo	0	Le Lamentin	Martinique	non	Pierre Damien Lucas
06/05/2014	MNHN JL146 + photo	1	Le Gros Morne	Martinique	MT602619	Clément Dromer
21/03/2015	photo	0	La Trinité	Martinique	non	Régis Delannoye
05/08/2015	MNHN JL262	1	La Trinité	Martinique	MT602622	Olivier Palcy
17/10/2015	photo	0	Le Gros Morne	Martinique	non	Pierre Damien Lucas
12/11/2015	MNHN JL305	1	Le Morne Vert	Martinique	MT602624 *	Mathieu Coulis
13/11/2015	photo	0	Ducos	Martinique	non	Cedric Rareg
04/02/2016	MNHN JL289	1	Le Marigot	Martinique	MT602623	Régis Delannoye
18/06/2017	MNHN JL310 + photo	1	Le Lamentin	Martinique	MT602625	Mathieu Coulis
22/06/2017	photo	0	Fort de France	Martinique	non	Marcel Bourgade
27/01/2018	photo	0	Sainte-Luce	Martinique	non	Stéphane Bras
12/02/2019	photo	0	La Trinité	Martinique	non	Régis Delannoye
20/12/2012	photo	0	Trois Rivières	Guadeloupe	non	Guy van Laere
06/12/2014	MNHN JL216 + photo	1	Gourbeyre	Guadeloupe	MT602620	Laurent Charles
12/12/2014	MNHN JL217 + photo	1	Bouillante	Guadeloupe	MT602621	Laurent Charles
21/01/2016	photo	0	Baillif	Guadeloupe	non	Pierre et Claudine Guezennec
20/12/2017	MNHN JL319 + photo	1	Trois Rivières	Guadeloupe	MT602626	Guy van Laere
06/08/2018	photo	0	Sainte-Anne	Guadeloupe	non	Jean-Christian Rotger

Références

Abascal F, Posada D, and Zardoya R. 2007. MtArt: a new model of amino acid replacement for Arthropoda. *Molecular Biology and Evolution* 24:1-5.

Álvarez-Presas M, Carbayo F, Rozas J, and Riutort M. 2011. Land planarians (Platyhelminthes) as a model organism for fine-scale phylogeographic studies: understanding patterns of biodiversity in the Brazilian Atlantic Forest hotspot. *Journal of Evolutionary Biology* 24:887-896.

Anonymous. 2020. La Liste rouge des espèces menacées en France. Faune de Martinique. Comité français de l'UICN, OFB & MNHN. *available from: https://inpnmnhnfr/docs/LR_FCE/Fascicule_liste_rouge_faune_martinique_2020_04_vfpdf (consulted 14 May 2020).*

Bankevich A, Nurk S, Antipov D, Gurevich AA, Dvorkin M, Kulikov AS, Lesin VM, Nikolenko SI, Pham S, and Prjibelski AD. 2012. SPAdes: a new genome assembly algorithm and its applications to single-cell sequencing. *Journal of Computational Biology* 19:455-477.

Bernt M, Donath A, Jühling F, Externbrink F, Florentz C, Fritzsch G, Pütz J, Middendorf M, and Stadler PF. 2013. MITOS: improved de novo metazoan mitochondrial genome annotation. *Molecular Phylogenetics and Evolution* 69:313-319.

Bowles J, Blair D, and McManus DP. 1995. A molecular phylogeny of the human schistosomes. *Molecular Phylogenetics and Evolution* 4:103-109.

Carbayo F, Álvarez-Presas M, Jones HD, and Riutort M. 2016. The true identity of *Obama* (Platyhelminthes: Geoplanidae) flatworm spreading across Europe. *Zoological Journal of the Linnean Society* 177:5–28.

Cuevas-Caballé C, Riutort M, and Álvarez-Presas M. 2019. Diet assessment of two land planarian species using high-throughput sequencing data. *Scientific Reports* 9:8679.

Delannoye R, Charles L, Pointier J-P, and Massemin D. 2015. *Mollusques continentaux de la Martinique*: Muséum national d'Histoire naturelle.

Gastineau R, and Justine J-L. 2020. Complete mitogenome of the invasive land flatworm *Parakontikia ventrolineata*, the second Geoplanidae (Platyhelminthes) to display an unusually long cox2 gene. *Mitochondrial DNA Part B* 5:2115-2116.

Gastineau R, Justine J-L, Lemieux C, Turmel M, and Witkowski A. 2019. Complete mitogenome of the giant invasive hammerhead flatworm *Bipalium kewense*. *Mitochondrial DNA Part B* 4:1343-1344.

Gastineau R, Lemieux C, Turmel M, and Justine J-L. 2020. Complete mitogenome of the invasive land flatworm *Platydemus manokwari*. *Mitochondrial DNA Part B* 5:1689-1690.

Gordon D, Abajian C, and Green P. 1998. Consed: a graphical tool for sequence finishing. *Genome research* 8:195-202.

Grau JH, Sluys R, Froehlich EM, and Carbayo F. 2012. Reflections on the genus *Amaga* Ogren and Kawakatsu 1990, and description of a new genus of land planarian (Platyhelminthes: Tricladida: Geoplanidae). *Journal of Natural History* 46:1529-1546.

Jones HD, and Sterrer W. 2005. Terrestrial planarians (Platyhelminthes, with three new species) and nemertines of Bermuda. *Zootaxa* 1001:31-58.

Justine J-L. 2019. Plathelminthes terrestres invasifs. Blog (in French). *https://sites.google.com/site/jljjustine/plathelminthe-terrestre-invasif*.

Justine J-L, Lemarcis T, Gerlach J, and Winsor L. 2018a. First report of the land planarian *Endeavouria septemlineata* (Hyman, 1939) (Platyhelminthes, Tricladida, Continenticola, Geoplanidae) in French Polynesia. *Zootaxa* 4450:297-300.

Justine J-L, Winsor L, Barrière P, Fanai C, Gey D, Han AWK, La Quay-Velazquez G, Lee BPY-H, Lefevre J-M, Meyer J-Y, Philippart D, Robinson DG, Thévenot J, and Tsatsia F. 2015. The invasive land planarian *Platydemus manokwari* (Platyhelminthes, Geoplanidae): records from six new localities, including the first in the USA. *PeerJ* 3:e1037.

Justine J-L, Winsor L, Gey D, Gros P, and Thévenot J. 2014. The invasive New Guinea flatworm *Platydemus manokwari* in France, the first record for Europe: time for action is now. *PeerJ* 2:e297.

Justine J-L, Winsor L, Gey D, Gros P, and Thévenot J. 2018b. Giant worms *chez moi*! Hammerhead flatworms (Platyhelminthes, Geoplanidae, *Bipalium* spp., *Diversibipalium* spp.) in metropolitan France and overseas French territories. *PeerJ* 6:e4672.

Justine J-L, Winsor L, Gey D, Gros P, and Thévenot J. 2019. *Obama chez moi!* The invasion of metropolitan France by the land planarian *Obama nungara* (Platyhelminthes, Geoplanidae). *PeerJ* 8:e8385.

Justine JL, and Winsor L. 2020. First record of presence of the invasive land flatworm *Platydemus manokwari* (Platyhelminthes, Geoplanidae) in Guadeloupe. *Preprints* 2020:2020050023.

Katoh K, and Standley DM. 2013. MAFFT multiple sequence alignment software version 7: improvements in performance and usability. *Molecular Biology and Evolution* 30:772-780.

Kumar S, Stecher G, and Tamura K. 2016. MEGA7: Molecular Evolutionary Genetics Analysis version 7.0 for bigger datasets. *Molecular Biology and Evolution* 33:1870-1874.

Lázaro EM, Sluys R, Pala M, Stocchino GA, Baguñà J, and Riutort M. 2009. Molecular barcoding and phylogeography of sexual and asexual freshwater planarians of the genus *Dugesia* in the Western Mediterranean (Platyhelminthes, Tricladida, Dugesiidae). *Molecular Phylogenetics and Evolution* 52:835-845.

Littlewood DTJ, Rohde K, and Clough KA. 1997. Parasite speciation within or between host species? - Phylogenetic evidence from site-specific polystome monogeneans. *International Journal for Parasitology* 27:1289-1297.

Lohse M, Drechsel O, Kahlau S, and Bock R. 2013. OrganellarGenomeDRAW—a suite of tools for generating physical maps of plastid and mitochondrial genomes and visualizing expression data sets. *Nucleic Acids Research* 41:W575-W581.

Lowe TM, and Chan PP. 2016. tRNAscan-SE On-line: integrating search and context for analysis of transfer RNA genes. *Nucleic Acids Research* 44:W54-W57.

Mateos E, Tudó A, Álvarez-Presas M, and Riutort M. 2013. Planàries terrestres exòtiques a la Garrotxa. *Annals de la Delegació de la Garrotxa de la Institució Catalana d'Història Natural* 6:67-73.

Ogren RE, and Kawakatsu M. 1990. Index to the species of the family Geoplanidae (Turbellaria, Tricladida, Terricola): Part I: Geoplaninae. *Bulletin of Fuji Women's College* 28:79-166.

Ross E, Blair D, Guerrero-Hernández C, and Alvarado AS. 2016. Comparative and transcriptome analyses uncover key aspects of coding-and long noncoding RNAs in flatworm mitochondrial genomes. *G3: Genes, Genomes, Genetics* 6:1191-1200.

Sakai M, and Sakaizumi M. 2012. The complete mitochondrial genome of *Dugesia japonica* (Platyhelminthes; order Tricladida). *Zoological science* 29:672-680.

Solà E, Álvarez-Presas M, Frías-López C, Littlewood DTJ, Rozas J, and Riutort M. 2015. Evolutionary analysis of mitogenomes from parasitic and free-living flatworms. *PLoS ONE* 10:e0120081.

Stamatakis A. 2014. RAxML version 8: a tool for phylogenetic analysis and post-analysis of large phylogenies. *Bioinformatics* 30:1312-1313.
